# The MAM’Out project: a randomized controlled trial to assess multiannual and seasonal cash transfers for the prevention of acute malnutrition in children under 36 months in Burkina Faso

**DOI:** 10.1186/s12889-015-2060-3

**Published:** 2015-08-08

**Authors:** Audrey Tonguet-Papucci, Lieven Huybregts, Myriam Ait Aissa, Jean-François Huneau, Patrick Kolsteren

**Affiliations:** Research and Analyses Department, Action Contre la Faim, Paris, France; Department of Food Safety and Food Quality, Ghent University, Ghent, Belgium; AgroParisTech, CRNH-IdF, UMR914 Nutrition Physiology and Ingestive Behavior, Paris, France; NRA, CRNH-IdF, UMR914 Nutrition Physiology and Ingestive Behavior, Paris, France; Poverty, Health and Nutrition Division, International Food Policy Research Institute, Washington, DC USA; Child Health and Nutrition Unit, Institute of Tropical Medicine, Antwerp, Belgium

**Keywords:** Cash transfer, Safety nets, Acute malnutrition, Wasting, Children, Burkina Faso, Research protocol

## Abstract

**Background:**

Wasting is a public health issue but evidence gaps remain concerning preventive strategies not primarily based on food products. Cash transfers, as part of safety net approach, have potential to prevent under-nutrition. However, most of the cash transfer programs implemented and scientifically evaluated do not have a clear nutritional objective, which leads to a lack of evidence regarding their nutritional benefits.

**Methods/Design:**

The MAM’Out research project aims at evaluating a seasonal and multiannual cash transfer program to prevent acute malnutrition in children under 36 months, in terms of effectiveness and cost-effectiveness in the Tapoa province (Eastern region of Burkina Faso, Africa). The program is targeted to economically vulnerable households with children less than 1 year old at the time of inclusion. Cash is distributed to mothers and the transfers are unconditional, leading to beneficiaries’ self-determination on the use of cash. The study is designed as a two-arm cluster randomized intervention trial, based on the randomization of rural villages. One group receives cash transfers via mobile phones and one is a control group. The main outcomes are the cumulative incidence of acute malnutrition and the cost-effectiveness. Child anthropometry (height, weight and MUAC) is followed, as well as indicators related to dietary diversity, food security, health center utilization, families’ expenses, women empowerment and morbidities. 24 h-food recalls are also carried out. Individual interviews and focus group discussions allow collecting qualitative data. Finally, based on a theory framework built a priori, the pathways used by the cash to have an effect on the prevention of under-nutrition will be assessed.

**Discussion:**

The design chosen will lead to a robust assessment of the effectiveness of the proposed intervention. Several challenges appeared while implementing the study and discrepancies with the research protocol, mainly due to unforeseen events, can be highlighted, such as delay in project implementation, switch to e-data collection and implementation of a supervision process.

**Trial registration:**

ClinicalTrials.gov, identifier: NCT01866124, registered May 7, 2013.

## Background

With at least 52 million wasted children in the world [1], wasting is a crucial public health issue. Although treatments for severely acute malnourished children exist and have proven their efficacy [2, 3], curative approaches remain very expensive [4] and more evidence is needed concerning strategies related to the management of moderate acute malnutrition [5]. The second Lancet Series on Maternal and Child Under-nutrition [5] and the Scaling-Up Nutrition Initiative [6] give some recommendations on selected effective approaches for the management and prevention of under-nutrition, such as breastfeeding counselling or micronutrient supplementation, but evidence gaps still remain, particularly concerning indirect interventions. The World Health Organization highlighted in 2010 the need to consider prevention strategies when implementing programs aiming at reducing acute malnutrition rates [7]. There is also evidence showing that preventive programs, such as supplementation, can be more effective to reduce childhood under-nutrition than nutrition rehabilitation [8]. Most scientific evaluations of nutrition rehabilitation are based on product distribution [9–13]. However, products are not always locally available nor affordable for the target population. Considering the paucity of data pertaining to alternative context-adapted strategies for the prevention of acute malnutrition, research studies must be developed in order to produce evidence on effective, reproducible and cost-effective approaches [14].

**Fig. 1:**
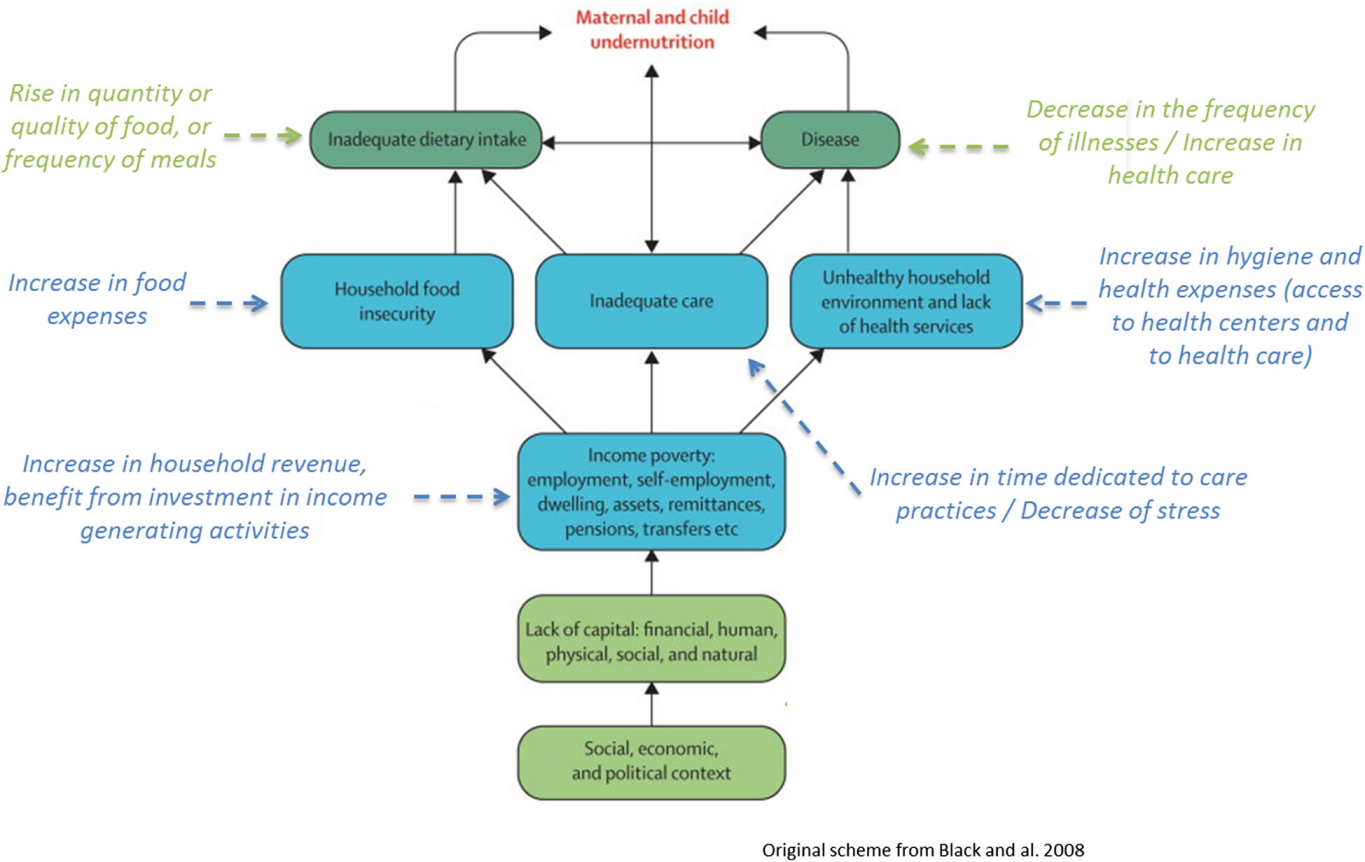
Proposed effects of cash transfers on the prevention of child undernutrition

Cash transfers, as part of a safety net approach, are relatively new in fragile states. Only a few safety net experiences for very poor and hunger vulnerable households have been implemented to date [15]. Indeed, humanitarian agencies have longstanding experiences with one-shot cash transfer interventions in emergency situations, but multiannual cash transfer is usually not implemented in countries exposed to acute malnutrition. Reviews on cash transfer experiences show that this type of intervention has the potential to prevent undernutrition [16, 17]. However, most of the cash transfer programs implemented and scientifically evaluated do not have a clear nutritional objective, which leads to inconclusive evidence regarding their nutritional benefits [18]. Hence, the MAM’Out (Moderate Acute Malnutrition Out) research project aims at assessing a context-adapted preventive approach, which is likely to influence several underlying causes of under-nutrition and not primarily based on food supplementation: seasonal and multiannual cash transfers. Indeed, as shown in Fig. 1, cash transfers can have an effect on all underlying causes of undernutrition. They have proven to be effective in removing financial barriers to health centers and nutritious food [19–21], especially in Latin America countries. Positive effects of cash transfer programs on poverty reduction and food security [22], diet quality [23] and child health [19, 20] have also been documented. Some reports also suggest benefices on maternal mental health [24]. One can also hypothesize that benefiting from cash transfers can allow mothers to reduce their income generating activities, leading to more time for child’s care. Most of the cited evidence comes from conditional cash transfers. However, the conditional aspect of cash transfer can be associated with several disadvantages or constraints [25] and was sometimes shown not to be appropriated, especially in African countries [26]. The MAM’Out project will thus evaluate the effects of unconditional cash transfers on the prevention of undernutrition.

Furthermore, there is a lack of evidence related to the pathways by which cash transfers can improve child nutrition outcomes. In their review’s conclusion, Gentilini and Omamo [27] highlight the need for more targeted study designs that could attribute effects to specific processes. From an intended impact pathways model, Adato and al [19] already evaluated qualitative field studies in middle income countries to explain why expected nutrition and health outcomes do or do not occur: poverty, sociocultural norms and beliefs on health care practices seem to compete with cash. This research will follow the proposition made by some authors [21, 28] to use a program theory framework to analyze the way in which different components interact in order to have an effect.

## Methods/Design

### Objectives of the research

The primary objective of the MAM’Out research project is to evaluate the effectiveness and cost-effectiveness of multiannual and seasonal cash transfers (MCTs) to prevent acute malnutrition in children under 36 months in the East region of Burkina Faso.

The specific objectives of this project are: 1. To measure the contribution of MCTs to the reduction of the incidence of acute malnutrition and morbidity for the young children; 2. To evaluate the input of MCTs in the young children’s growth and development; 3. To contribute to the creation of an evidence base on efficient preventing activities for child wasting; 4. To assess the influence of MCTs on determinants of acute malnutrition, such as food security and access to health center; 5. To evaluate the cost-effectiveness of MCTs for the prevention of acute malnutrition in order to improve their sustainability.

### Study population

The target populations are inhabitants of the Eastern region of Burkina Faso, and more specifically the Tapoa province, where Gourmanche people are in the majority. This population faces the highest fertility rate in the country, with 8.6 children per woman [29]. This leads to small interpregnancy intervals that negatively impact maternal nutritional status, leading to poor birth outcome. Results of a cross-sectional survey (SMART) aiming at estimating malnutrition prevalence in the Tapoa province in April 2012 also showed a prevalence of global acute malnutrition of 17.3 % (95 %CI: 15.2 – 19.7) among children aged 6 to 59 months (2006 WHO growth references). In this context, the MAM’Out research project specifically targets households with children under 12 months at the time of inclusion.

Thirty two (32) villages, situated in the northern part of the Tapoa province and belonging to the same livelihood zone are included in the study. Villages were selected within three municipalities, based on their geographic localization and other operational criteria, such as accessibility.

To be included in the study, households had to meet two criteria: to be classified as poor or very poor according to the Household Economy Approach [30] and to have at least one child under 1 year old at the time of inclusion, independently of his/her nutritional status. All households present in the selected villages and meeting both criteria were included in the study.

### Intervention

The proposed approach is based on monthly seasonal cash transfers for 5 months, from July to November, and for two years (2013 and 2014). A monthly 10 000 FCFA is transferred to the selected households. The amount was determined during a cash assessment study implemented in collaboration with Action Contre la Faim operational team in Burkina Faso in October 2012 and with reference to other experiences of cash transfers in the Sahel area [31]. Mothers, as the primary responsible for children’s health and nutrition, are the recipient of the cash transfer. In order to avoid destabilizing the family organization or leading to a misuse of the money, the project includes a sensitization strategy for heads of household, mothers-in-law and important people in the villages on the objectives of the project and the reasons of the choice of women as cash recipient. Cash transfers are done via mobile phones, in collaboration with a private mobile phone company chosen according to ethical criteria defined by Action Contre la Faim’s procurement department. This is a quite innovative method in Burkina Faso, as the first mobile payment system was developed only in July 2012 in the country. This way of transfer was chosen for practical and security reasons: it represents much less risks for the staff and beneficiaries. Mobile phones and SIM cards are provided to mothers.

Besides, in order to reduce the risk of drop out in the control group, compensations for the time people spent for the project are offered to all participants of this group.

### Study design and randomization

The study is designed as a two-arm clustered randomized controlled trial in the Tapoa province (Eastern region of Burkina Faso), with one group benefiting from cash transfers and one being a comparison group. The unit of randomization is the village. Subjects are assigned to the study groups according to where they live. The random assignment of the interventions is done through a ceremonial gathering with officials and community members, as well as a representative of each of the concerned villages (mainly the heads of the village). Thirty-two (32) papers with a word corresponding to one group (“cash” for the intervention group and “pas cash” for the control group) are put in a bag. Each representative of the 32 villages is asked to draw blindly from the bag a paper. The village is allocated to one of the groups according to the word on the drawn paper.

### Recruitment and informed consent

A first oral agreement is sought collectively at the community level before the beginning of the study. A representative of each village involved in the study (mainly the heads of villages and their committees) is asked to give his consent for the village participation into this research, as suggested by the WHO CIOMS Guidelines [32]. Once this first acknowledgement received, a second informed consent is sought individually. Before being enrolled in the study, heads of households and mothers are explained the aim of the research, the expected duration of their participation and the measurements that will be done. According to allocated group, each participant also receives a global explanation on the sequence of the activities and procedures. Risks and benefits associated with his/her participation are also presented. Families that refuse consent are not forced into the study because of the collective agreement. As the major part of the population is illiterate, care is taken to give all these explanations orally in the local language. Written inform consent is sought individually by a local officer part of the research team before the beginning of the study. Mothers who agree to take part in the research are asked to write down their name or make a mark with their finger print. In the second case, a second research officer or the head of the village is asked to witness the process.

### Data collection

Data collection is performed quarterly for two years by trained staff under the supervision of a field study coordinator. All participants of the study are visited every three months at home and asked to answer various questions. The collected data is immediately coded. None of the paper records includes the child’s name or address. Each field staff member has a separate register in which the correspondence between the name and address of children and the unique identification number (UIN) of children is made. In addition, heads of families are given a trial card containing information about their name and address as well as their UIN. This is to ensure that records can be accessed even in case of accidental destruction of the registers. Missing data is defined as being absent during two consecutive three-monthly visits. In such case, a home visit is organized to document the reason of the absence.

### Model theory framework

A nutritional causal analysis was conducted in the Tapoa province in November-December 2012. In addition to the already available data and context analyses, this survey allowed defining more deeply the pathways by which cash transfers can have an effect on acute malnutrition according to the local context. A model theory framework was then built and was the basis for the choice of most of the indicators followed during the study.

### Outcome measures

The primary outcomes of the study are the cumulative incidence of child wasting and the incremental cost-effectiveness ratio. Secondary outcomes are the cumulative incidence of the state of stunting, mean height-for-age Z-score, mean weight-for-length Z-score, mid-upper arm circumference (MUAC), edema, as well as rates of diarrhea, acute respiratory infections and measles.

**Fig. 2:**
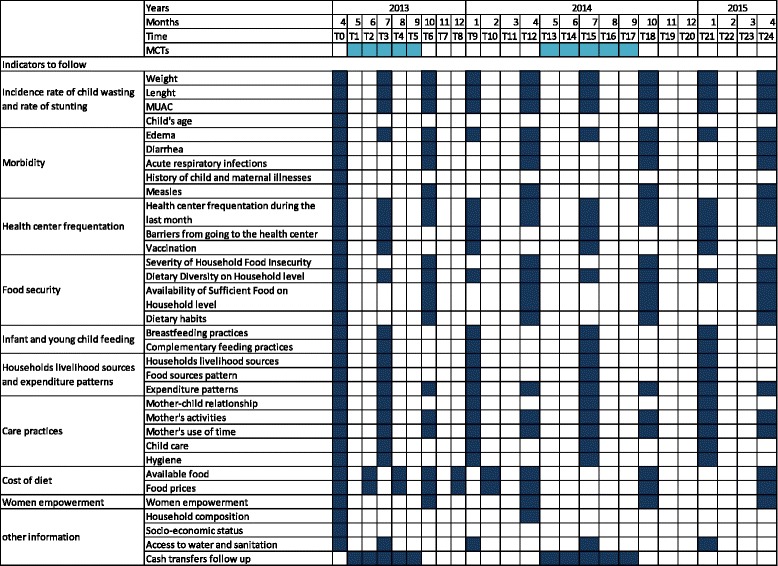
Indicators and chronogram of measurement as initially forecasted in the research protocol

Upon inclusion, the mother is interviewed to obtain baseline information including household composition, socio-economic status, dietary habits, child’s age, breastfeeding practices and a history of child and maternal illnesses. Intermediate factors such as food security, water access, dietary diversity scores, mother-child relationship, women’s role and health center frequentation are also asked for. During the follow up, the same information collected at inclusion time is collected again at different time points. All questionnaires were translated in local language during the training session of the data collection staff, back translated in French and pretested locally. Figure 2 summarizes the quantitative data collection throughout the two years of the study. Two 24 h-food recalls are also planned as part of children nutritional assessment.

Qualitative data is also collected via focus group discussions and individual interviews. Systematic focus groups are organized in all villages of the intervention group. The primary aim is to allow participants to exchange experiences of usage of the cash transfer which is part of the intervention program. Simultaneously these focus groups offer a possibility to evaluate the hypothesized action theory model of cash transfers to prevent acute malnutrition. A semi-structured questionnaire is used to assess the experiences related to all possible cash pathways. All discussions are recorded on tape. Observations made during the interviews are also reported.

### Measurement instruments

The child’s weight is recorded using an electronic mother-child weighting scale (Model 876, SECA, Germany). Length is recorded to the nearest 1 mm using foldable length boards (Model 417, SECA, Germany). MUAC is recorded using a non-stretchable plastic tape (model 201, SECA, Germany). All measurements are taken in duplicate during each home visit.

Research teams also interview for disease episodes during the last week using a standardized and tested questionnaire (acute respiratory infections, diarrhea, fever, malnutrition, malaria and measles). Diarrhea is defined as “the passage of 3 or more loose or liquid stools per day” (WHO definition). The symptoms detailed by Roth [33] are used to define acute respiratory infections: “At least one lower respiratory tract sign reported by a caregiver and/or observed by study personnel (fast or difficulty breathing, chest wall indrawing) and/or abnormal findings on pulmonary auscultation (crackles/crepitation and/or bronchial breath sounds)”. Fever is defined as a temperature superior or equal to 38 °C.

### Standardization procedure

Data collectors are trained in all procedures in order to minimize the bias linked to the data collection officers. Standardization exercises for anthropometric measures and interview techniques are organized before the beginning of the study. Questionnaires are standardized and pre-tested. Animators of the focus groups are also trained by qualified people, so that all discussion groups and individual interviews are handled in the same way.

### Sample size calculation

In order to detect a decrease with 33 % in the cumulative incidence of wasting assuming a baseline incidence rate of wasting of 0.26 per child-year [9] with a Type I error of 5 %, a statistical power of 90 % and a minimum follow-up time of 24 months, assuming a coefficient of variation K of 0.25, we calculated that 16 clusters of 50 households per cluster are necessary per study arm [34]. This calculation takes into account an anticipated 25 % drop-out.

### Statistical and qualitative analyses

Descriptive analysis will compare changes in endpoints and intermediate indicators between study groups. Cumulative incidence of wasting will be analyzed using mixed-effects Poisson regression accounting for the clustered design by village. The hypothesized change in mean weight-for-height/weight-for-length z-score will be analyzed using a linear mixed model accounting for clustering by village (random intercept). The addition of a random slope (per child) to the analysis model will be tested using a restricted maximum likelihood ratio test. Models will be adjusted for important covariates related to the child wasting incidence, namely child’s sex, child’s age, baseline nutritional status and household socio-economic score, to gain precision of model estimates. In addition, if important baseline imbalances are noticed, a sensitivity analysis will be conducted adding these imbalances to the aforementioned models. To assess the influence of missing data, a sensitivity analysis will be carried using a multiple imputation strategy to account for missing data.

Cumulative incidence of stunting and morbidity will be analyzed using mixed-effects Poisson regression accounting for the clustered design. Continuous outcomes like mean length-for-age/height-for-age z-scores, mean length/height, mean weight, mean MUAC, will be analyzed using linear mixed models adjusted for child’s sex, age and baseline condition of the outcome of interest. As a sub-analysis we will analyze the intermediate endpoints, ie after the first intervention period (2013) and the second intervention period (2014).

Cost-effectiveness will be evaluated through the calculation of cost-effectiveness ratios in terms of cost per new case of acute malnutrition averted (thanks to the cash transfer), and incremental differences in costs and outcomes between intervention and control groups [35, 36]. This will allow for the evaluation of the program effect. The measurement of total cost to achieve outcomes will be done through the ABC (Activity Based Costing) approach: ingredient costs are grouped by “cost centers” based on activities and support costs are allocated to activities based on activity time allocation from staff interviews. A separate protocol was developed in order to detail all the procedures related to the calculation of the cost-effectiveness.

Qualitative analyzes will also be conducted with data from focus groups and individual meetings with women. All recorded audio will be transcribed and translated in French into a Word document. Data obtained from these discussions will be coded using NVIVO 10.0 software. The coding will be performed by pathway corresponding to the interview guide, but also using an iterative method to integrate emerging pathways (open coding). This will allow modifying and/or validating the model theory framework constructed a priori and working on the pathways taken by cash transfers in order to have an impact of the prevention of acute malnutrition. Moreover, explorative pathway analysis will be conducted to identify the most important changes in intermediate covariates responsible for the hypothesized change in the outcome (incidence of wasting). Mediation analysis/pathway analysis will finally be performed to identify in a quantitative manner the most important pathways through which the hypothesized change in primary outcomes is mediated.

### Ethical considerations

The protocol was submitted to two independent ethics committees. The study was approved in April 2013 by the Ethical Committee of the University Hospital of Ghent and in May 2013 by the Burkinabe National Ethical Committee. Official documents are available on requests. This study is also registered in ClinicalTrials.gov: NCT01866124 since May 7, 2013.

### Collaborating organizations

This study is implemented by Action Contre la Faim – France, with the scientific support of Ghent University (Belgium), the Institute of Tropical Medicine Antwerp (Belgium), AgroParisTech (France), the Center for Disease Control (Unites States of America) and the Institut de Recherche en Sciences de la Santé (Burkina Faso). It is funded by Action Contre la Faim – France and the Center for Disease Control. The cash transfer program was made possible thanks to ECHO funds. The cost-effectiveness analysis is co-funded by Action Contre la Faim and the Nutrition Embedding Evaluation Program (NEEP, PATH-DFID).

## Discussion

The MAM’Out research project is a two-arm cluster randomized controlled trial aiming at assessing the effectiveness and the cost-effectiveness of seasonal and multiannual cash transfers to prevent acute malnutrition in Burkinabe children under 36 months.

Studies implemented in humanitarian situations are often merely observational with mostly a pre vs. post evaluation [37] and thus do not allow for a robust assessment of the effectiveness of the implemented activities. The design chosen here will lead to an evidence-based evaluation of the proposed intervention. The presence of a control group seems acceptable as the activities implemented are preventive and not curative ones. Moreover, the children included in the control group benefit from a regular and intensive follow-up allowing for an early detection of acute malnutrition cases. In such events, children are referred and managed by the nearest health center supported by Action Contre la Faim. Additionally, efforts are made to the collect high quality data on intermediate process parameters (such as food security or access to health facilities) which will allow for the identification and understanding of the changes activated during cash transfer programs.

This study has been implemented in the field since June 2013. Up to date care has been taken to rigorously follow the research protocol. However some discrepancies mainly due to unforeseen events can be highlighted. First, waiting for ethical clearances, the project faced a two-month delay compared to the timeline presented in Fig. 2: the baseline measurements started in June instead of April 2013. This led to a two-month postponement of the beginning of the cash transfers which are therefore implemented from July to November. Secondly, after one year of project implementation, there was a switch from a standard paper data collection to e-data collection via tablets. This solution responds to delays in data entry and allows for real time follow up of the data collected. Thirdly, a supervision process not described initially in the research protocol has been implemented. It ensures the quality of data collection and homogeneity between the four groups of data collectors. Finally, an evaluation of the cost of a local and balanced diet according to the season (cost of the diet) was planned in the protocol. This study won’t be carried out but the price of the major staple food is monthly followed in the field.

Several challenges may still arise regarding the implementation of the study. However, to stick to the nine rounds of data collection planned in the research protocol, the end of the project is forecasted for September 2015. With a design based on a cluster randomized controlled trial, this study will lead to a strong evaluation of the effects of multiannual and seasonal cash transfers for the prevention of children acute malnutrition.

## Abbreviations

FCFA: Franc of the Financial Community in Africa
MAM’Out: Moderate Acute Malnutrition Out
MCTs: Multiannual and seasonal Cash Transfers
MUAC: Mid-Upper Arm Circumference
UIN: Unique Identification Number
WHO: World Health Organization

## References

[1] Black RE, Victora CG, Walker SP, Bhutta ZA, Christian P, de Onis M, Ezzati M, Grantham-McGregor S, Katz J, Martorell R (2013). Maternal and child undernutrition and overweight in low-income and middle-income countries. Lancet.

[2] Bhutta ZA, Ahmed T, Black RE, Cousens S, Dewey K, Giugliani E, Haider BA, Kirkwood B, Morris SS, Sachdev HP (2008). What works? Interventions for maternal and child undernutrition and survival. Lancet.

[3] WHO (2013). Guideline: Updates on the Management of Severe Acute Malnutrition in Infants and Children.

[4] Webb P, Rogers B, Rosenberg IH, Schlossman N, Wanke C, Bagriansky J, Sadler K, Johnson Q, Tilahun J, Masterson AR (2011). Delivering improved nutrition: recommendations for changes to US food aid products and programs.

[5] Bhutta ZA, Das JK, Rizvi A, Gaffey MF, Walker N, Horton S, Webb P, Lartey A, Black RE (2013). Evidence-based interventions for improvement of maternal and child nutrition: what can be done and at what cost?. Lancet.

[6] ScalingUpNutrition: A Framework For Action. http://scalingupnutrition.org/wp-content/uploads/pdf/SUN_Framework.pdf 2011.

[7] WHO, UNICEF, WFP, UNHCR (2010). Consultation on the Programmatic Aspects of the Management of Moderate Acute Malnutrition in Children under five years of age.

[8] Ruel MT, Menon P, Habicht JP, Loechl C, Bergeron G, Pelto G, Arimond M, Maluccio J, Michaud L, Hankebo B (2008). Age-based preventive targeting of food assistance and behaviour change and communication for reduction of childhood undernutrition in Haiti: a cluster randomised trial. Lancet.

[9] Isanaka S, Nombela N, Djibo A, Poupard M, Van Beckhoven D, Gaboulaud V, Guerin PJ, Grais RF (2009). Effect of preventive supplementation with ready-to-use therapeutic food on the nutritional status, mortality, and morbidity of children aged 6 to 60 months in Niger: a cluster randomized trial. Jama.

[10] Parikh K, Marein-Efron G, Huang S, O'Hare G, Finalle R, Shah SS (2010). Nutritional status of children after a food-supplementation program integrated with routine health care through mobile clinics in migrant communities in the Dominican Republic. Am J Trop Med Hyg.

[11] Huybregts L, Houngbe F, Salpeteur C, Brown R, Roberfroid D, Ait-Aissa M, Kolsteren P (2012). The effect of adding ready-to-use supplementary food to a general food distribution on child nutritional status and morbidity: a cluster-randomized controlled trial. PLoS Med.

[12] Hendricks KM (2010). Ready-to-use therapeutic food for prevention of childhood undernutrition. Nutr Rev.

[13] Imdad A, Sadiq K, Bhutta ZA (2011). Evidence-based prevention of childhood malnutrition. Curr Opin Clin Nutr Metab Care.

[14] Cattaneo A, Timmer A, Bomestar T, Bua J, Kumar S, Tamburlini G (2008). Child nutrition in countries of the Commonwealth of Independent States: time to redirect strategies?. Public Health Nutr.

[15] Fan S (2010). Halving Hunger: meeting the first millennium development goal through business as unusual.

[16] Leroy JL, Ruel M, Verhofstadt E, Olney D (2008). The micronutrient impact of multisectoral programs focusing on nutrition: examples from conditional cash transfer, microcredit with education and agricultural programs. Innocenti review.

[17] Attanasio O, Gómez LC, Heredia P, Vera-Hernández M. The short term impact of a conditional cash subsidy on child health and nutrition in Colombia. The Institute of Fiscal Studies. Report summary 2005, Familias 03:15 pp.

[18] Ruel MT, Alderman H (2013). Nutrition-sensitive interventions and programmes: how can they help to accelerate progress in improving maternal and child nutrition?. Lancet.

[19] Adato M, Bassett L (2009). Social protection to support vulnerable children and families: the potential of cash transfers to protect education, health and nutrition. AIDS Care.

[20] Lagarde M, Haines A, Palmer N (2009). The impact of conditional cash transfers on health outcomes and use of health services in low and middle income countries. Cochrane Database Syst Rev.

[21] Leroy JL, Ruel M, Verhofstadt E (2009). The impact of conditional cash transfer programmes on child nutrition: a review of evidence using a programme theory framework. Journal of Development Effectiveness.

[22] Fiszbein A, Schady N, Ferreira FHG, Grosh M, Kelleher N, Olinto P, Skoufias E (2009). Conditional Cash Transfers - Reducing present and future poverty. A World Bank Policy Research Report.

[23] Wall J, Mhurchu CN, Blakely T, Rodgers A, Wilton J (2006). Effectiveness of monetary incentives in modifying dietary behavior:a review of randomized, controlled trials. Nutr Rev.

[24] Fenn B, Noura G, Sibson V, Dolan C, Shoham J. The role of unconditional cash transfers during a nutritional emergency in Maradi region, Niger: a pre-post intervention observational study. Public Health Nutr. 2014;1–9. 10.1017/S1368980014000378 PMC10271468 24679647

[25] Gaarder M (2012). Conditional versus unconditional cash: a commentary. Journal of Development Effectiveness.

[26] Davis B, Gaarder M, Handa S, Yablonski J (2012). Evaluating the impact of cash transfer programmes in sub-Saharan Africa: an introduction to the special issue. Journal of Development Effectiveness.

[27] Gentilini U, Omamo SW (2011). Social protection 2.0: Exploring issues, evidence and debates in a globalizing world. Food Policy.

[28] Gaarder MM, Amanda G, TJ E (2010). Conditional cash transfers and health: unpacking the causal chain. Journal of Development Effectiveness.

[29] Dakuyo LM, Ouedraogo FG, Somda S (2009). Recensement général de la population et de l’habitation de 2006. Analyse des résultats définitifs. Thème 6 : natalité - fécondité.

[30] Boudreau T, Lawrence M, lzmann PH, O’Donnell M, Adams L, Holt J, Hammond L, Duffield A (2008). The Practitioners' Guide to the Household Economy Approach - Regional Hunger and Vulnerability Programme.

[31] Kauffmann D. Étude de faisabilité et définition de la méthodologie d’intervention en transferts sociaux au Burkina Faso. Action Contre la Faim Novembre. 2012.

[32] CIOMS (2002). International Ethical Guidelines for Biomedical Research Involving Human Subjects.

[33] Roth DE, Richard SA, Black RE (2010). Zinc supplementation for the prevention of acute lower respiratory infection in children in developing countries: meta-analysis and meta-regression of randomized trials. Int J Epidemiol.

[34] Hayes RJ, Bennett S (1999). Simple sample size calculation for cluster-randomized trials. Int J Epidemiol.

[35] Drummond MF, Sculpher MJ, Torrance GW, O'Brien BJ, Stoddart GL (2005). Methods for the economic evaluation of health care progammes.

[36] Muenning P. Cost-Effectiveness Analysis in Health: A Practical Approach. Jossey-Bass Inc. US 2007, 2nd Revised edition

[37] Bailey S, Hedlund K (2012). The impact of cash transfers on nutrition in emergency and transitional contexts. A review of evidence.

